# Nervonic acid in infant nutrition: a forward-looking approach to enhancing neurodevelopmental outcomes

**DOI:** 10.3389/fnut.2025.1635266

**Published:** 2025-06-26

**Authors:** Frédéric Destaillats, Manuel Oliveira, Walter Rakitsky, Xiaoying Zhou, Leon Parker

**Affiliations:** Checkerspot, Inc., Alameda, CA, United States

**Keywords:** nervonic acid, infant nutrition, myelination, milk-fat globule membranes, premature infants, fermentation, brain development, sphingolipids

## Abstract

Nervonic acid (24:1 n-9, NA) is a monounsaturated very long-chain fatty acid (VLCFA) that plays a fundamental role in brain development, particularly in the biosynthesis of sphingolipids and myelin sheaths. NA is present in minute amounts in human milk and despite its importance in neuronal function and cognitive development, there is currently no ingredient available for the fortification of infant nutrition products. However, recent advances in biotechnology have made it feasible to produce high NA containing oil through fermentation, presenting a significant opportunity to address this nutritional gap. This review explores the potential of NA fortification in infant nutrition products and its impact on neurodevelopment, with a specific focus on two populations: premature infants, who are at higher risk of neurodevelopmental impairments due to incomplete *in utero* myelination, and healthy term infants, who may experience enhanced cognitive development with improved dietary NA intake when consuming infant formula. By critically examining the scientific basis for NA supplementation, as well as the practical challenges and regulatory considerations associated with its implementation, this review aims at providing a forward-looking perspective on how this emerging ingredient could enhance infant nutrition and improve health outcomes.

## Introduction

The first year of life is a critical period of rapid brain growth and neural development, during which fundamental processes such as myelination, synaptogenesis, and neurotransmitter synthesis occur at an accelerated rate, shaping cognitive and sensory functions (1). Proper brain maturation and function during one's lifespan requires a precise balance of nutrients to support essential physiological mechanisms (2–7, 96). Studies have shown that specific nutrients, including phospholipids and sphingomyelin from milk fat globule membrane (MFGM), enhance myelination and cognitive development, underscoring the importance of dietary exposure in early infancy (1, 8). Additionally, brain iron levels play a vital role in key neurodevelopmental processes, as iron is essential for neurotransmitter synthesis and myelination, with research showing a developmental coupling between brain iron and neural activity during the first 150 days of life (9).

Beyond structural development, functional brain connectivity in early infancy has a lasting impact on cognitive abilities in later childhood (10, 11). Research has found that early growth trajectories influence the development of functional connectivity, which is associated with cognitive flexibility at preschool age, highlighting how undernutrition or adverse early environments can negatively impact neural network maturation (12). Furthermore, language acquisition and sensory processing abilities undergo significant refinement in this period, as evidenced by studies demonstrating the emergence of cortical phonetic feature encoding in infants between 4 and 11 months of age, providing neurophysiological proof that early phonetic categorization shapes future language development (13). Similarly, thalamocortical circuit development, which governs sensory integration and higher cognitive functions, follows distinct age-dependent trajectories in infancy, reinforcing the idea of early-life neuroplasticity as a determinant of long-term brain function (14, 15). Collectively, these findings emphasize that the first year of life represents a critical window for neural development, where nutritional, environmental, and genetic factors interact to shape lifelong cognitive and behavioral outcomes.

Lipids are particularly vital during this stage, as they constitute nearly 60% of the brain's dry weight and play fundamental roles in neuronal membrane integrity, fluidity, and function (16, 17). Among these, VLCFAs are of special interest due to their involvement in the synthesis of sphingolipids, which are key structural components of neural membranes and myelin sheaths (18). Myelination, the process of forming the protective sheath around neurons, is essential for efficient brain signaling and cognitive development. One of the most important VLCFA in this context is nervonic acid (NA; 24:1 n-9), a unique fatty acid predominantly found in sphingomyelin. The presence of sphingolipids in human breast milk plays a crucial role in early-life brain development, as they provide essential building blocks for membrane structure and neurodevelopmental functions (19, 20). Studies have shown that infants fed with MFGM containing infant formula, a rich source of phospholipids and sphingomyelin, exhibit enhanced brain myelination and improved cognitive abilities, particularly in non-verbal and fine motor domains (1, 8).

In this review, we explore the potential of NA fortification in infant nutrition products and its impact on neurodevelopment, with a particular focus on two key populations: premature infants, who are at higher risk of neurodevelopmental impairments due to incomplete *in utero* myelination, and healthy term infants, who may experience enhanced cognitive development with improved dietary NA intake. Additionally, we review the levels of NA in human milk and propose appropriate fortification levels for infant nutrition products to better align with physiological needs and support optimal neurodevelopment. Furthermore, we examine the natural sources of NA and highlight promising research efforts aimed at developing new dietary sources through fermentation of genetic engineered microbes.

## The biochemistry and physiological role of nervonic acid in early life

During pregnancy, the placenta plays a critical role in selectively transporting VLCFA such as long-chain polyunsaturated fatty acids (LC-PUFAs), particularly docosahexaenoic acid (DHA) and arachidonic acid (ARA), to the fetus to support neurodevelopment. This selective transfer is facilitated by specialized transport proteins, including plasma membrane fatty acid-binding proteins (p-FABPpm) and fatty acid transport proteins (FATPs), which mediate the uptake of DHA and ARA from maternal circulation (21). Additionally, the major facilitator superfamily domain-containing 2a (MFSD2a) transporter facilitates the transfer of these fatty acids esterified to lysophosphatidylcholine, rather than as free fatty acids (22). While these mechanisms are well-documented for DHA and ARA, current scientific literature does not provide strong evidence that the placenta prioritizes NA and further research is needed to elucidate the placental transport mechanisms of NA and its potential role in fetal neurodevelopment. The study by Bettger and co-workers demonstrated that NA from the maternal diet is efficiently transferred to milk during lactation in a rodent model (23). Dietary supplementation with NA-rich oils led to increased levels of NA in the sphingomyelin fraction of milk, which was subsequently absorbed by suckling rat pups. This transfer resulted in changes in the sphingomyelin composition of the heart and liver in the offsprings, indicating that dietary NA is bioavailable through milk and can be incorporated into developing tissues (23).

**Figure 1:**
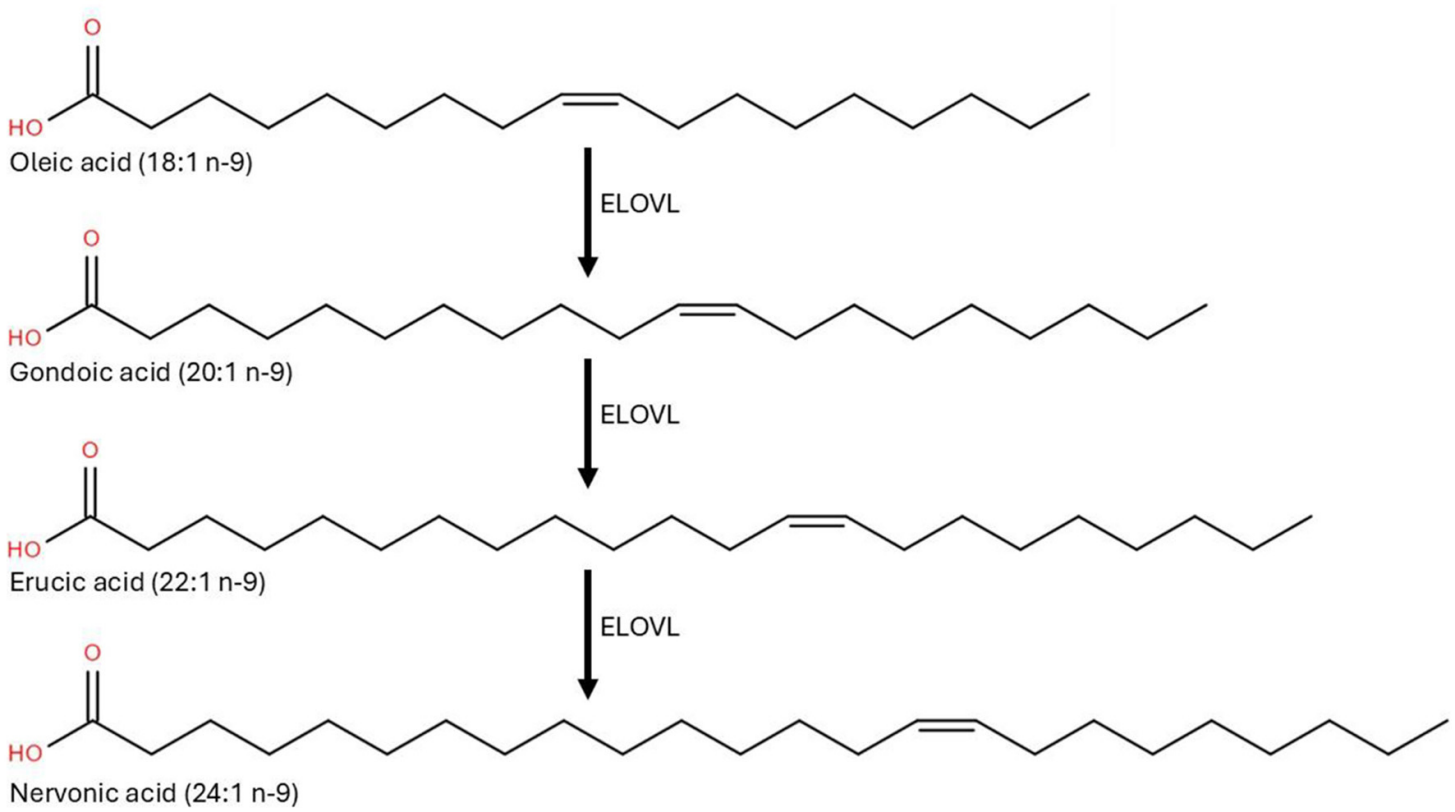
Schematic representation of the elongation of oleic (18:1 n-9) to nervonic (24:1 n-9) by the elongation of very long-chain fatty acid enzymes system [ELOVL; adapted from Garcia Corrales et al. (25)].

**Figure 2:**
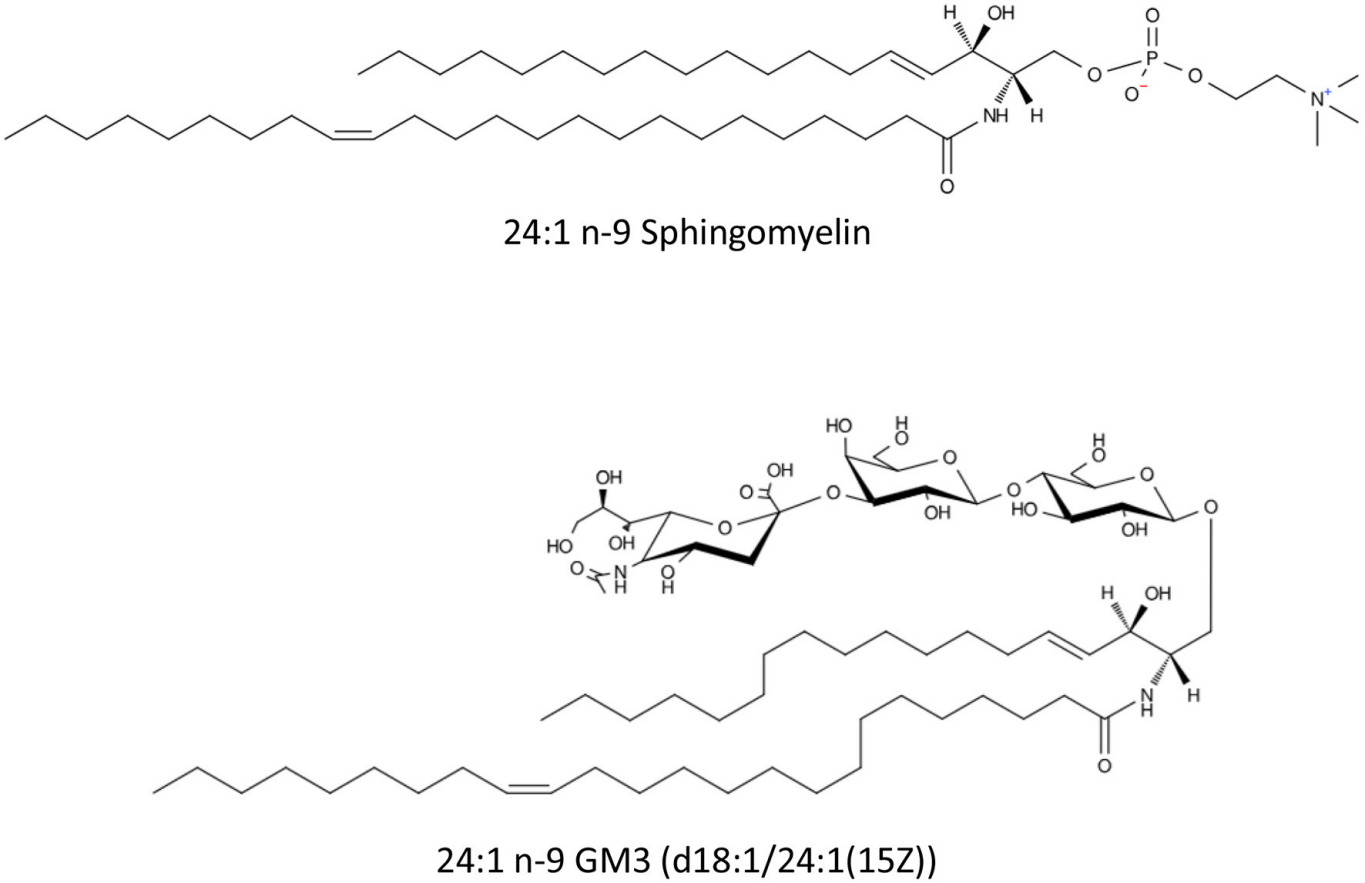
Schematic representation of nervonic acid containing sphingomyelin [N-(15Z-tetracosenoyl)-sphing-4-enine-1-phosphocholine] and ganglioside GM3 [NeuAcα2-3Galβ1-4Glcβ-Cer(d18:1/24:1(15Z))].

During fetal development and infancy, NA supports the rapid expansion of neural networks, the formation of myelin, and the structural integrity of cellular membranes, processes critical for cognitive and motor function maturation (24). NA biosynthesis is particularly active in the liver and brain during early life (see Figure 1), driven by the elongation of oleic (18:1 n-9), gondoic (20:1 n-9) and erucic (22:1 n-9) acids (25). Studies indicate that NA supplementation can enhance myelin synthesis in oligodendrocytes, promoting nerve maturation (26). Once produced, NA is incorporated into sphingomyelin and gangliosides (Figure 2) that are major lipid constituent of the myelin sheath that insulates axons, enabling rapid and efficient nerve impulse conduction (24). NA facilitates proper signal transduction and cell recognition, contributing to optimal neural connectivity and long-term neurodevelopmental outcomes (24). Unlike shorter-chain fatty acids that primarily serve as energy substrates, NA is integral component of cellular membranes, particularly in the nervous system, where they contribute to membrane fluidity, stability, and signaling (24, 27).

The critical role of NA in neurodevelopment is underscored by its association with disorders characterized by defective myelination. Inherited peroxisomal disorders, such as Zellweger syndrome and X-linked adrenoleukodystrophy (X-ALD), involve disruptions in VLCFA metabolism, leading to the accumulation of toxic saturated VLCFA and deficits in myelination (24). These conditions often manifest with severe neurodevelopmental impairments, progressive demyelination, and loss of motor and cognitive function, highlighting the indispensable role of VLCFA like NA in maintaining neuronal structure and function (24).

## Evolution of the concentration of nervonic acid in human milk along the lactation stages

In the present review, we evaluate the concentration of NA in human breast milk all along different lactation stages, infant populations (term vs. preterm), and geographic regions based on a comprehensive literature search which has been compiled in Table 1. It is important to note that only a fraction of studies reporting the fatty acid profile of human milk provide data on NA concentrations. The reason for this omission is not entirely clear, especially considering that NA, when analyzed as a methylester derivative, elutes in the same gas-chromatographic region as DHA, a fatty acid that has been reported in >50 studies over the past 30 years. Nevertheless, we were able to gather data from 24 studies, collectively representing 2,404 human milk samples collected across 15 countries (see Table 1). Across all studies, the concentration of NA is highest in colostrum (average: 0.39% of total fatty acids), followed by transitional milk (0.18%) and mature milk (0.11%). The highest NA content in colostrum (0.72%) was observed in samples from Turkey (28), while the lowest (0.19%) was found in samples from Sudan (29).

**Table 1:** Level of nervonic acid in colostrum, transitional milk (trans. Milk) and mature milk reported in 24 published studies, collectively representing 2,404 human milk samples collected across 15 countries.

**References**	**Country/site**	**Samples**	**Concentration of nervonic acid in milk**		
			**Colostrum**	**Trans. milk**	**Mature milk**
Aydin et al. (28)	Turkey (Preterm)	15	0.72	0.77	0.64
Aydin et al. (28)	Turkey	15	0.78	0.74	0.37
Giuffrida et al. (34)	China (Guangzhou)	180	0.40	0.10	0.10
Giuffrida et al. (34)	China (Beijing)	179	0.40	0.10	0.10
Giuffrida et al. (34)	China (Suzhou)	180	0.40	0.10	0.10
Yu et al. (30)	China (Wuxi)	224	0.20	0.15	0.06
Thakkar et al. (39)	Switzerland (Preterm)	279	0.30	0.13	0.07
Thakkar et al. (39)	Switzerland	219	0.39	0.13	0.07
Yakes et al. (80)	Bangladesh	98			0.20
Li et al. (43)	China (Guangzhou)	25		0.06	0.06
Li et al. (43)	China (Shanghai)	25		0.08	0.05
Li et al. (43)	China (Nanchang)	25		0.12	0.11
Li et al. (43)	China (Harbin)	11		0.06	0.04
Li et al. (43)	China (Hohhot)	11		0.21	0.19
Golfetto et al. (81)	Korea (Karen, Camp 1)	36			0.05
Golfetto et al. (81)	Korea (Karen, Camp 2)	53			0.07
Golfetto et al. (81)	Korea	12		0.27	
Duan et al. (42)	Korea	34			0.06
Mihályi et al. (82)	Hungaria	87	0.26		0.06
Peng et al. (83)	China (Changzhou)	82	0.45		
Peng et al. (83)	China (Wenzhou)	20	0.25		
Nyuar et al. (29)	Sudan	60	0.19	0.15	0.02
Zhao et al. (84)	China (Beijing)	50	0.54		0.25
Young et al. (85)	Japan	36	0.40		
Knox et al. (86)	Nigeria	89			0.07
Sala-Vila et al. (87)	Spain	30		0.04	0.05
Moltó-Puigmartí et al. (41)	Spain (very preterm)	10	0.21	0.10	0.06
Moltó-Puigmartí et al. (41)	Spain (preterm)	10	0.30	0.11	0.05
Moltó-Puigmartí et al. (41)	Spain	23	0.32	0.08	0.05
Xiang et al. (88)	Sweden	19	0.54	0.11	
VanderJagt et al. (89)	Nigeria	34			0.07
Marangoni et al. (90)	Italia	10	0.31		0.13
Marangoni et al. (91)	Italia	22	0.36		0.12
Xiang et al. (92)	China	41			0.15
Rueda et al. (40)	Spain (preterm)	6	0.37	0.27	
Rueda et al. (40)	Spain	16	0.44	0.18	0.08
Rocquelin et al. (93)	Congo	102			0.04
Boersma et al. (94)	St Lucia	36	0.41	0.11	0.04
Average			0.39	0.18	0.11
Median			0.39	0.11	0.07
SD			0.15	0.19	0.12

Results are expressed in % of the total fatty acids.

**Figure 3:**
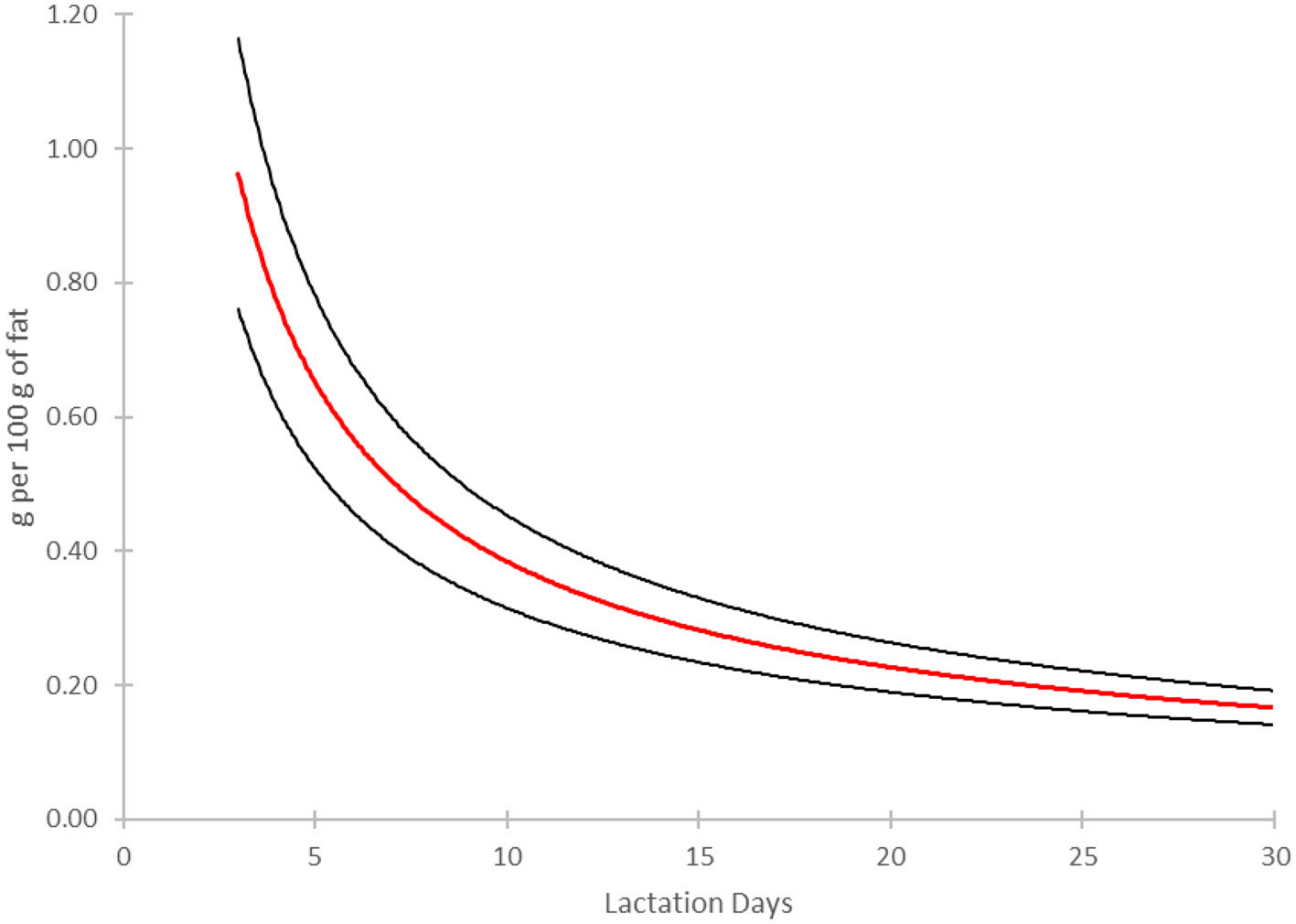
Longitudinal evolution of the concentration of nervonic acid (24:1 n-9, NA) in human milk over the course of the first month of lactation. The model has been built based on the original data (30). The red line represents a power regression (y = 2.2229·x^−0.762^) fitted to 12 data points describing the concentration of NA (g per 100 g of fat) as a function of lactation days. The regression yielded a high coefficient of determination (R^2^ = 0.996), indicating an excellent fit. Black lines indicate the mean ± 1 standard deviation of the fitted values.

To further illustrate the decline of NA levels across lactation stages, a model was constructed based on the data from published longitudinal study (30). Figure 3 presents a modeling of the evolution of the NA concentration, expressed in mg of NA per g of fat in human milk, over the first month of lactation (adapted from 28). The model confirms a consistent decline in NA concentration over the first 30 days of lactation (Figure 3). The red curve represents the modeled mean trend, while the black curves indicate confidence intervals (+/- 1 standard deviation, Figure 3). Similar observations has been reported in a collection of studies performed in Hungary (31).

The decline of NA levels in breast milk observed over time (Figure 3) likely reflects the progressive maturation of the metabolic pathways involved in its *de novo* synthesis in growing infants, paralleling other developmental shifts in nutrient availability and metabolism. As a key component of myelin, NA plays a crucial role in white matter development during early brain growth (27). At birth, infants rely on maternal milk as a primary source of fatty acids, including NA, to support rapid neural development. However, as enzymatic systems involved in fatty acid elongation and desaturation mature, endogenous synthesis of NA becomes increasingly efficient. This trend mirrors the developmental regulation of enzymes such as very long-chain fatty acid enzymes system type 4 (ELOVL4), which facilitates the elongation of VLCFA, peaking around birth and declining as the brain reaches a steady metabolic state (32). The shift in NA synthesis is analogous to the decline in breast milk protein content over time, which coincides with the slowing of postnatal growth velocity (33). Early in life, higher protein levels in breast milk accommodate the rapid growth demands of the neonate, but as growth rate decreases, so does the protein concentration in milk (33). Similarly, the synthesis of DHA, another critical fatty acid for neural development, follows a comparable trajectory (34), increasing as infants mature and their endogenous enzymatic pathways for fatty acid elongation and desaturation become more active. The competition between substrates for desaturases and elongases, including those shared between NA and omega-3/-6 fatty acid pathways, further suggests that dietary lipid availability influences the timing and efficiency of NA synthesis (35). Additionally, the transition from dependence on breast milk-derived NA to endogenous production aligns with broader metabolic maturation, including improvements in lipid digestion and assimilation mechanisms, such as increased bile salt availability and pancreatic lipase activity (36–38).

A comparison of milk secreted from mother who delivered term and preterm infants revealed no significant differences in NA concentrations at various lactation stages. Thakkar and co-workers reported NA levels of 0.30% in colostrum, 0.13% in transitional milk, and 0.07% in mature milk for preterm infants (39), which closely matched the values observed in term infants (0.39%, 0.13%, and 0.07%, respectively). Similarly, Rueda and co-workers found slightly higher transitional milk NA levels in preterm infants (0.27%) compared to term infants (0.18%), but the overall trend remained consistent, indicating that preterm birth does not significantly impact NA concentrations (40). Same observations have been made in studies conducted in Spain (41), Poland (95), and Turkey (28). In conclusion, NA levels are highest in colostrum and gradually decrease as lactation progresses, with no significant differences observed between term and preterm infants. While regional variations exist, they do not appear to affect the general trend. These findings underscore the importance of early NA provision for neonatal neurodevelopment and highlight the potential for NA supplementation in infant nutrition products.

**Figure 4:**
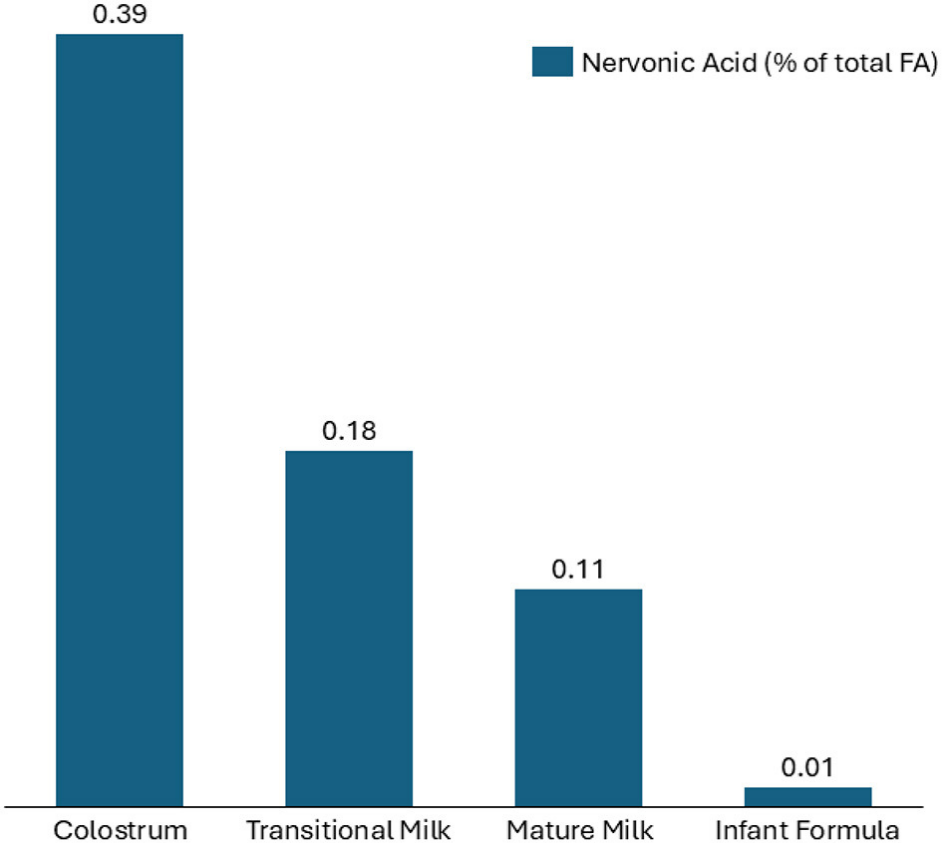
Comparison of the level of nervonic acid (24:1 n-9) in colostrum, transitional, mature human milk (data reported in Table 1) and in infant formula (42, 43). Data are expressed in % of total fatty acids.

In contrast, the concentration of NA in infant formula (42, 43) is substantially lower than in human milk, as illustrated in Figure 3. Colostrum exhibits the highest NA content (0.39% of total fatty acids), followed by transitional milk (0.18%) and mature milk (0.11%), while infant formula contains only 0.01% (Figure 4). This considerable difference suggests that formula-fed infants receive markedly lower NA levels than their breastfed counterparts. Given NA's role in neural membrane formation and myelination, its limited presence in infant formula raises concerns regarding potential neurodevelopmental implications. These findings highlight the need for further research into NA fortification in infant nutrition products to better align with the composition of human milk.

## Potential benefits of nervonic acid fortification for premature infants

Premature birth, defined as delivery before 37 weeks of gestation, is associated with an increased risk of neurodevelopmental disorders, primarily due to disrupted brain maturation during the third trimester, a critical period for myelination and white matter development (44, 45). During this stage, oligodendrocyte proliferation, differentiation, and myelin deposition occur rapidly, facilitating efficient neural conductivity and cognitive development (46, 47). However, preterm infants miss the crucial placental transfer of DHA and ARA, which play fundamental roles in neuronal membrane integrity, synapse formation, and neural signaling (27, 48).

Clinical studies have demonstrated that low levels of sphingomyelin and VLCFA in preterm infants are associated with cognitive and motor impairments, underscoring the importance of these lipids for early brain development (27, 49). Sphingomyelin, a key component of the myelin sheath, relies on the availability of VLCFA such as NA for its synthesis (27, 50). Insufficient VLCFA supply in preterm infants has been linked to disruptions in myelin formation and white matter injury, increasing the risk of neurodevelopmental conditions, including cerebral palsy, attention-deficit/hyperactivity disorder (ADHD), and global developmental delays (46, 47).

Given these concerns, NA supplementation in preterm infants represents a promising strategy to support the postnatal myelination process. Adequate NA intake may enhance oligodendrocyte function, promoting myelin production and improving neural conductivity, potentially mitigating the long-term neurodevelopmental consequences of premature birth. Current best practices in neonatal intensive care units (NICUs) advocate for feeding premature infants with human milk from either the mother or hospital milk banks, as human milk provides optimal nutrition and immunological benefits essential for infant development (51). However, to meet the specific nutritional needs of preterm infants, human milk supplied to premature infants often requires fortification, particularly with proteins and lipids, to support adequate growth and development (52). When looking at the relatively low level of NA in mature milk (Table 1, Figure 3), it might be beneficial to fortify human milk used with additional level of NA to reach at least the high concentration observed in colostrum which is in the range of 1mg per g of fat in fortified human milk or synthetic formulation (Figure 3).

## Potential benefits of nervonic acid fortification for healthy term infants

Numerous studies in pediatric nutrition have demonstrated that early dietary interventions with nutritional lipids, such as DHA and sphingolipids in the form of MFGM, are associated with improved cognitive outcomes, higher IQ scores, and enhanced attention and problem-solving abilities later in childhood (1, 8, 53). Given that NA is a key component of infant neural tissues sphingolipids, its inclusion in infant formulas could provide an additional pool for their biosynthesis promoting neurodevelopmental advantage, potentially enhancing learning, memory retention, and overall brain function. During early life, NA is provided *in utero* and postnatally through human milk (Table 1, Figure 3). If breastfeeding is not possible, infant formulas are the preferred substitute. However, published studies have shown that current infant formulas do not contain nutritionally relevant amounts of NA (Figure 3), suggesting that fortification with a suitable dietary source of NA at physiological level of ca. 0.10–0.20 mg per g of fat may be a potential solution.

Although direct clinical trials on NA supplementation in infants are currently lacking, growing evidence from studies on sphingomyelin-enriched diets suggests that increasing dietary VLCFA could contribute to improved neurodevelopmental outcomes (8). Dietary SM, a sphingolipid containing VLCFA, has been associated with enhanced white matter maturation, faster information processing speeds, and better motor coordination in infants. For instance, a study by Tanaka and co-workers demonstrated that very low birth weight infants fed SM-fortified milk exhibited improved neurobehavioral development during infancy (54). Similarly, it has been reported that higher dietary SM intake in early life was linked to increased myelination and cognitive performance in children (55). These findings highlight the potential of VLCFA, including NA, to support neurodevelopment and cognitive function in early life, warranting further research to establish optimal dietary strategies for brain health.

## Natural sources of nervonic acid and their limitations in food applications

Although NA is naturally present in trace amounts across various plant and animal sources, the concentrations are insufficient for use in the fortification of products. For instance, low-erucic acid (22:1 n-9, EA), rapeseed oil, commercially known as canola oil, typically contains around 0.1% EA and a comparable level of NA. One of the richest known natural sources of NA is the seed oil of *Acer truncatum*, commonly known as the Shantung maple (56, 57). Native to East Asia, this tree produces seeds with oil containing ~4–8% NA, making it an attractive candidate for industrial extraction (56, 57). Other plant sources include the seeds of the malania (*Malania oleifera*) tree (58) and the cruciferous plant *Lunaria annua* (honesty plant), both of which also contain significant levels of NA (57). It has been recently demonstrated that the purification of NA as a methyl ester is possible by combining distillation and crystallization (59). In this study, a four-stage vacuum distillation process was optimized to enrich NA methyl ester from *Acer truncatum* seed oil, achieving 91% purity, followed by low-temperature crystallization to further purify NA to 98% (59). This complex process may only be cost-effective for the preparation of pure NA as an active pharmaceutical ingredient (API). In addition to plant-based sources, certain fish oils and animal-derived lipids, particularly those from marine organisms and ruminant fats, have been identified as dietary sources of NA. However, the overall dietary contribution of NA from these sources is relatively low, limiting their feasibility for large-scale nutritional applications.

Despite their high NA content, many of these oils also contain excessive levels of EA, a structurally similar VLCFA that has been associated with adverse health effects (see Table 2). Bettger and Blackadar (60) found that dietary EA had minimal impact on the 24:1/24:0 sphingomyelin ratio, unlike NA, which markedly increased it. This suggests EA is more readily degraded than incorporated into structural lipids as confirmed in a later study (61) who reported that 22:1n-9 underwent tissue selective metabolism, with conversion to 18:0 the dominant pathway in the liver presumably for export in the neutral lipids, while in heart it was found primarily as 22:1n-9 in neutral lipids and used for β-oxidation. Furthermore, EA's inefficient oxidation leads to the accumulation of metabolic intermediates, particularly in the heart, where peroxisomal activity is lower (23). Prolonged EA exposure has been associated with myocardial lipidosis, characterized by triglyceride accumulation within cardiac muscle fibers, impairing mitochondrial oxidative metabolism (62). Cardiac lesions, including necrosis and fibrosis, have been observed in male Sprague-Dawley rats fed high-EA rapeseed oil (63). Additionally, EA suppresses long-chain fatty acid oxidation, exacerbating lipid accumulation and interfering with normal cardiac energy metabolism (62). Structural alterations, including intracellular lipid droplet accumulation and lysosomal involvement in triglyceride hydrolysis, may further contribute to its cardiotoxic effects (64). While these toxic effects are well-documented in animal models, human evidence remains inconclusive, necessitating further research. In contrast, NA is efficiently utilized for sphingolipid synthesis in the heart, reducing the risk of excessive accumulation and associated toxicity (23). As a result, stringent regulatory limits have been imposed on its presence in food products. The U.S. Food and Drug Administration (FDA) have established strict guidelines on a maximum of 2% of EA in edible oils (Code of Federal Regulation: 21 CFR 184.1555). The authors did not find any studies reporting harmful effects of high doses of nervonic acid (NA) in either preclinical models or clinical trials.

**Table 2:** Oil, nervonic (24:1 n-9) and erucic (22:1 n-9) acids content in some selected seed oil.

**Reference**	**Plant**	**Oil content (%)**	**Nervonic acid (% of total FA)**	**Erucic acid (% of total FA)**
Liu et al. (57)	*Acer truncatum Bunge*	45–48	3.9–7.8	≈17.0
Liu et al. (57)	*Melania oleifera Chun*	58–63	56.0–67.0	≈13.0
Liu et al. (57)	*Lunaria annual*	25–35	14.0–24.2	43.0–50.0
Bettger et al. (23)	*Tropaeolum speciosum*	Not reported	38.7	13.3
Li et al. (70)	*Xanthoceras sorbifolium*	Not reported	3.8	8.5

Due to these safety concerns, traditional plant sources of NA are generally unsuitable for direct inclusion in food formulations. This challenge has led to increasing interest in biotechnological alternatives, such as fermentation technologies, which offer a scalable, sustainable solution for the nutrition and health industries (65).

## Production of nervonic acid in transgenic plants and microorganisms

Biosynthesis of NA has emerged as a promising alternative to conventional extraction methods, providing a sustainable and potentially high-yield approach to meet growing market demands. It has been recently reviewed in great details (65–67) and this section critically examines advancements and challenges associated with NA biosynthesis through transgenic plants and microorganisms.

Transgenic plants have long been studied for NA production due to their naturally high oil content and well-established genetic modification protocols. Initial efforts in the late 20^th^ century were aimed at inserting elongase genes from various species into oilseed crops to boost NA synthesis. One pioneering study demonstrated that introducing an elongase gene into rapeseed significantly enhanced NA yields, establishing proof-of-concept for NA biosynthesis *via* transgenic approaches (68). Subsequent research successfully transferred the KCS gene from *Lunaria annua* (69) and *Xanthoceras sorbifolium* (70) to *Arabidopsis thaliana*, resulting in an increase in EA and NA content. Another notable study involved the introduction of the KCS gene from *Cardamine graeca* into Brassica species, which substantially boosted NA production and emphasized the critical importance of selecting suitable donor genes and recipient plant species (71). In this study, the highest NA level in transgenic *B. carinata* lines reached 44%, with only 6% of residual of EA (71). Recent research confirmed the high elongation activity of the MoKCS11 gene from *Malania oleifera* when expressed in *Arabidopsis thaliana* and *Camelina sativa*, thereby demonstrating its versatility and significant potential for broader application in agricultural biotechnology (72).

Microorganisms, particularly yeasts, have been engineered for NA production due to their rapid growth rates, scalability, and ease of cultivation under controlled conditions. Initial success in microbial NA and EA synthesis was reported in *Saccharomyces cerevisiae* by overexpressing genes encoding KCS and elongases, marking a significant milestone in microbial production capabilities (70, 73). The potential for producing NA using genetically engineered *Yarrowia lipolytica* has been previously investigated and reviewed (74). Further advancements have led to the development of *Y. lipolytica* strain capable of producing NA at 22–23% and gondoic acid (20:1 n-9, GA) at 8–10% of total fatty acids, providing a strong foundation for future optimization efforts (75). Further work on *Y. lipolytica* strains has explored multiple strategies in parallel, including the overexpression of genes involved in the desaturation and TAG assembly, leading to several noteworthy findings (76, 77). Additional work involved coexpression of the KCS genes from *Crambe abyssinica* and *C. graeca* in *Rhodosporidium toruloides*, significantly enhancing NA and EA concentrations, 7.9% and 5.8% of total FA, respectively in a 7L fermentation trial (78). To our knowledge, the best composition that has been achieved to date in a yeast framework has been obtained in *R. toruloides* by integrating KCS gene and optimization of the metabolic pathway (79) allowing to reach a concentration of NA of 46.3% and of EA of 2.8% of total fatty acids. While the composition obtained in the study is extremely interesting, the productivity (44.2 g/L in a 5 L bioreactor.) obtained remains a concern for commercial production.

Significant advancements have been made in biosynthesis across transgenic plants, microorganisms, and microalgae; however, considerable challenges such as improving yield and productivity in fermentation operations and reducing cultivation costs remain. Future research should emphasize optimizing genetic engineering, fermentation conditions, and cultivation practices to enhance NA production efficiency, yield, and sustainability, thus addressing critical market demands and supporting sustainable nutritional strategies.

## Challenges and considerations in implementing nervonic acid fortification

Despite the promising potential of NA in infant nutrition, several challenges must be addressed before its widespread implementation. One major hurdle is regulatory approval, as novel food ingredients require extensive clinical validation to ensure safety and efficacy. Additionally, the stability of NA in infant formula formulations must be optimized to prevent oxidation and maintain bioavailability. Furthermore, ethical and economic considerations must be considered to ensure equitable access to NA-fortified products, preventing disparities in infant nutrition. As research progresses, collaborations between the scientific community, regulatory agencies, and industry stakeholders will be crucial in overcoming these barriers and bringing NA-fortified formulas to market.

## Conclusion

The integration of NA into infant nutrition products represents an exciting opportunity for pediatric nutrition, with the potential to enhance neurodevelopmental outcomes in both premature and term infants. As biotechnology continues to progress, the sustainable microbial production of NA-rich oils will facilitate its inclusion in infant formulas, bridging a critical gap in lipid nutrition. Future research should focus on establishing optimal NA dosage, assessing long-term neurodevelopmental benefits, and addressing regulatory challenges to ensure the safe and effective implementation of NA fortification in infant nutrition.
